# The complete chloroplast genome of *Artocarpus heterophyllus* (Moraceae)

**DOI:** 10.1080/23802359.2017.1413317

**Published:** 2017-12-12

**Authors:** Jin Liu, Ying-Feng Niu, Shu-Bang Ni, Zi-Yan Liu, Cheng Zheng, Chao Shi

**Affiliations:** a Yunnan Institute of Tropical Crops, Xishuangbanna, China;; b Yunnan Branch of Institute of Medicinal Plant Development, Chinese Academy of Mecidal Science, Xishuangbanna, China;; c Kunming Institute of Botany, Chinese Academy of Sciences, Kunming, China

**Keywords:** Chloroplast genome, *Artocarpus heterophyllus*, Moraceae

## Abstract

The *Artocarpus heterophyllus,* native to Western Ghats of India, Malaysia and south-eastern Asia, is a tree member of the mulberry family (Moraceae). Chloroplast genome sequences play a significant role in the development of molecular markers in plant phylogenetic and population genetic studies. In this study, we report the complete chloroplast genome sequence of *A. heterophyllus* for the first time. The chloroplast genome is 160,387 bp long and includes 113 genes. Its LSC, SSC and IR regions are 88,422, 18,869 and 26,548 bp long, respectively. Phylogenetic tree analysis exhibited that *A. heterophyllus* was clustered with other Moraceae species with high bootstrap values.

The *Artocarpus heterophyllus* is a species of the mulberry family (Moraceae), and it is native to Western Ghats of India, Malaysia and south-eastern Asia (Rahman et al. 1999). It is a large, evergreen fruit tree, with 10–15 m in height, indigenous to the evergreen forests at altitude of 450–1200 m and bear the largest known edible fruit (up to 35 kg). There have been five complete chloroplast genomes reported representing three genus in the family Moraceae, but none of them belongs to the genus *Artocarpus*. In this study, we report the complete chloroplast genome of *A. heterophyllus*, and hope it would aid further phylogenetic and population genetic studies of the *Artocarpus* species.

DNA material was isolated from mature leaves of an *A. heterophyllus* plant cultivated in the plant garden of Yunnan Institute of Tropical Crops (YITC), Jinghong, China by using DNeasy Plant Mini Kit (QIAGEN, Hilden, Germany). A specimen of this tree was conserved in YITC. About 10 μg isolated DNA was sent to BGI, Shenzhen for library construction and genome sequencing on the Illumina Hiseq 2000 Platform (Illumina, San Diego, CA). After genome sequencing, a total of 3.4 Gbp reads in fastq format were obtained and subjected to chloroplast genome assembly. The complete chloroplast genome was annotated with Dual Organelle GenoMe Annotator (DOGMA; Wyman et al. 2004) and submitted to the Genbank under the accession number of MG434693.

Our assembly of the *A. heterophyllus* resulted in a final sequence of 160,387 bp in length with no gap. The overall A-T content of the chloroplast genome was 61.2%. This chloroplast genome included a typical quadripartite structure with the large single copy (LSC), small single copy (SSC) and inverted repeat (IR) regions of 88,422, 18,869 and 26,548 bp long, respectively. Genome annotation showed 113 full-length genes including 79 protein-coding genes, 30 tRNA genes and four rRNA genes. The genome organization, gene content and gene relative positions were almost identical to the previously reported Moraceae chloroplast genomes. Eighteen genes were duplicated in the IR regions. Fifteen genes contained one intron, while three had two introns. Phylogenetic tree was constructed with the complete chloroplast genomes of *A. heterophyllus* and the other five representative species in Moraceae. Maximum-likelihood (ML) analysis exhibited that *A. heterophyllus* was clustered with other Moraceae species with high bootstrap values (Figure 1). The evolutionary relationships of these analyzed species are consistent with previously reported results (Zerega et al. 2005).

**Figure 1.**
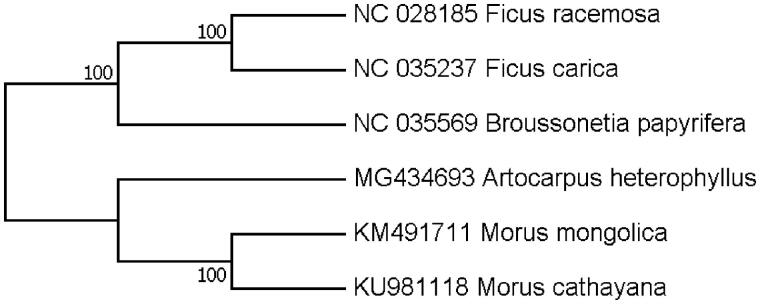
Maximum-likelihood (ML) phylogenetic tree of *A. heterophyllus* and the other five representative Moraceae species. Number above each node indicates the ML bootstrap support values.

## References

[CIT0002] Rahman AM, Nahar N, Mian AJ, Mosihuzzaman M. 1999 Variation of carbohydrate composition of two forms of fruit from jack tree (Artocarpus heterophyllus L) with maturity and climatic conditions. Food Chem. 65:91–97.

[CIT0003] Wyman SK, Jansen RK, Boore JL. 2004 Automatic annotation of organellar genomes with dogma. Bioinformatics. 20:3252–3255. 15180927 10.1093/bioinformatics/bth352

[CIT0004] Zerega NJ, Clement WL, Datwyler SL, Weiblen GD. 2005 Biogeography and divergence times in the mulberry family (Moraceae). Mol Phylogenet Evol. 37:402–416. 16112884 10.1016/j.ympev.2005.07.004

